# Descriptive study of the oral health status of disadvantaged mexican populations in relation to their adherence to the mediterranean diet

**DOI:** 10.21142/2523-2754-1202-2024-196

**Published:** 2024-06-27

**Authors:** Javier Flores-Fraile, Sergio Parra-García, Diego González-Gil, Alejandro Moreno-Barrera, Alejandra Peramato-Benito, Antonio Castaño-Seiquer

**Affiliations:** 1 Department of Surgery, Faculty of Medicine. University of Salamanca, Salamanca, Spain. j.flores@usal.es , sergiopg@usal.es , diegoggil@usal.es , alejandraperamato@usal.es Universidad de Salamanca Department of Surgery Faculty of Medicine University of Salamanca Salamanca Spain j.flores@usal.es sergiopg@usal.es diegoggil@usal.es alejandraperamato@usal.es; 2 Department of Stomatology, Faculty of Odontology Universidad de Sevilla, Sevilla, Spain. almobar92@hotmail.com , acastano@us.es Universidad de Sevilla Department of Stomatology Faculty of Odontology Universidad de Sevilla Sevilla Spain almobar92@hotmail.com acastano@us.es

**Keywords:** oral epidemiology, obesity, oral health, diet, preventive dentistry, prevention, Mediterranean diet, Yucatan, epidemiología bucal, obesidad, salud bucal, dieta, odontología preventiva, prevención, dieta mediterránea, Yucatán

## Abstract

Background: Obesity in Mexico is an alarming problem that has been increasing in recent decades. Dietary factors make this pathology more common at younger ages and closely related to oral health. This study attempts to investigate the association between the oral health status of a Mexican population in the state of Yucatan and their dietary habits. Objective: This study explores the relationship between oral health-related quality of life and adherence to the Mediterranean diet in a disadvantaged population in the state of Yucatan, Mexico. Methods: The research was conducted in July 2023 in Merida, Yucatan (Mexico). The sample consisted of 109 individuals aged between 4 and 72 years old. Data analysis focused on factors such as body mass index (BMI), oral health-related quality of life, and adherence to the Mediterranean diet. Results: A notable presence of caries is observed in individuals with low adherence to the Mediterranean diet (Correlation coefficient 0.040, p=0.682). This underscores the potential interaction between oral health, obesity, and dietary habits. The mean Oral Health-Related Quality of Life (OHIP-14Sp) score was 13.19 ±13.57, median 8.00. Conclusions: This research adds to the increasing evidence that highlights the significance of a balanced diet in enhancing the oral quality of life for people. More research is necessary to explore preventive measures and treatment to raise awareness about oral health within the community.

## RESUMEN

Antecedentes: La obesidad en México es un problema alarmante que ha ido en aumento en las últimas décadas. Los factores dietéticos hacen que esta patología sea más común en edades más tempranas y muy relacionada con la salud bucal. Este estudio intenta investigar la asociación entre el estado de salud bucal de una población mexicana en el estado de Yucatán y sus hábitos alimentarios. Objetivo: Este estudio explora la relación entre la calidad de vida relacionada con la salud bucal y la adherencia a la dieta mediterránea en una población desfavorecida del estado de Yucatán, México. Métodos: La investigación se realizó en julio de 2023 en Mérida, Yucatán (México). La muestra estuvo compuesta por 109 individuos con edades comprendidas entre 4 y 72 años. El análisis de los datos se centró en factores como el índice de masa corporal (IMC), la calidad de vida relacionada con la salud bucal y la adherencia a la dieta mediterránea. Resultados: Se observa una notable presencia de caries en individuos con baja adherencia a la dieta mediterránea (coeficiente de correlación 0,040, p=0,682). Esto subraya la posible interacción entre la salud bucal, la obesidad y los hábitos alimentarios. La puntuación media de la calidad de vida relacionada con la salud bucal (OHIP-14Sp) fue 13,19 ± 13,57, mediana 8,00. Conclusiones: Esta investigación se suma a la creciente evidencia que resalta la importancia de una dieta equilibrada para mejorar la calidad de vida bucal de las personas. Es necesaria más investigación para explorar medidas preventivas y tratamientos para crear conciencia sobre la salud bucal dentro de la comunidad.

## INTRODUCTION

Oral health is a dynamic state influenced not only by hygiene and dietary habits, but also by the absence of oral and systemic diseases ^1^^,^^2^. Currently, oral cavity diseases present a high prevalence and incidence, significantly impacting the perceived quality of life and daily functioning of individuals due to factors such as pain, inflammation, and functional limitations ^3^^-^^6^.

Over 70% of the population presently experiences some form of oral pathology, defined as deviations from ideal oral health, including complete dentition, absence of pathology, proper dental and intermaxillary relationships, and optimal morpho-functional parameters, all of which can variably affect systemic health. This prevalence is notably higher in underdeveloped or developing countries despite basic dental care being provided by the National Health System ^6^^,^^7^. In Mexico, for example, epidemiological data suggests a prevalence of oral pathology exceeding 90%, reflecting both a local and broader socio-health issue. This research specifically focuses on the Yucatan region, serving as a microcosm whose oral and systemic pathology statistics can be extrapolated nationally ^8^^-^^11^.

Numerous oral diseases stem from unhealthy dietary habits, with the Mediterranean diet serving as a reference model ^12^^-^^18^. These oral diseases present significant public health challenges, resulting in pain, social consequences, and functional limitations. Globally, nine out of ten individuals are at risk of experiencing such issues ^19^^-^^23^.

Mexico is among countries with a high prevalence of oral diseases, with caries affecting over 90% of its population. It is important to note that the Universal Catalog of Health Services (CAUSES) provides medical coverage, including dental specialties ^24^^,^^25^.

Focusing on the Yucatan region, the General Directorate of Epidemiology data indicates oral disease rates in 2019 comparable to national averages and significantly lower than those observed in other North American or European nations ^26^.

Obesity in Mexico is a growing concern, driven by socio-cultural and dietary factors, despite governmental efforts to promote healthy lifestyles and their impact on quality of life. This issue is closely related to oral health, as optimal oral health facilitates efficient initial digestive processes such as chewing, bolus formation, and swallowing ^27^^-^^29^.

This study aims to investigate the association between oral health status and adherence to the Mediterranean diet in a disadvantaged Mexican population in the state of Yucatan.

## MATERIAL AND METHODS

### Type of study and characteristics.

An observational, cross-sectional, and descriptive study was conducted as part of the "Odontólogo Residente Internacional" course at two health centers in the city of Mérida, in the state of Yucatán, Mexico, in July 2023.

The study adhered to the principles outlined in the Declaration of Helsinki and obtained approval from an Ethics Committee (protocol code 03/2018, approved in August of 2018). Informed consent was obtained from all participants, as well as from parents or legal guardians if applicable. The study followed STROBE guidelines.

The sample comprised 109 patients who attended routine check-ups at public health centers without prior knowledge of the data collection to prevent bias. Participants ranged in age from 4 to 72 years. They voluntarily sought dental and medical care and were provided detailed oral explanations before signing an informed consent form. Subsequently, they completed a research dossier covering questions about their oral and dental health, medical history, adherence to a Mediterranean diet, body measurements, and socioeconomic status, although the latter was not directly relevant to the investigation's primary objective.

All patients underwent examination by the same clinical examiner using a consistent methodology to minimize bias. Data collection was performed by a single experienced dentist specializing in prosthesis assessment. Intra-observer calibration was conducted to assess the consistency of observations, yielding a Kappa test agreement ratio of 0.85.

### Measurement of variables

The study examined the following variables: gender, body mass index (BMI), oral quality of life, and the presence of dental caries. Gender was the main variable of interest.

The diet was evaluated using an oral quality of life and nutritional habits questionnaire OHIP-14Sp.

These variables were collected during voluntary dental exams at the "International Resident Dentist" campaign site. The same examiner recorded and transcribed the data to create a validated research dossier.

### Statistical analysis. 

The qualitative and quantitative variables were analyzed using the statistical software NCSS277/GESS2006. Descriptive and inferential statistics were be employed, including cross-tabulation analysis, Fisher's exact test, Chi-square test, Student's t-test, Pearson correlation test, and multivariate analysis. A significance level of p < 0.05 was accepted as statistically significant.

All data was collected in a Microsoft Excel v.16 (2020) database for subsequent analysis.

## RESULTS

### Age

The mean age was 35.53 years, SD ±19.58, median 37.00, range 4-72.

Table 1 displays the age in the study groups, with no significant differences found (p=0.544).

**Table 1:** Age in the study groups.

Group	Mean	SD	Median	Range
Female Group	39.95	18.81	38.00	4-72
Male Group	34.38	21.85	32.00	5-66

#### 3.2. Body Mass Index.

The mean body mass index (BMI) was 29.16, SD ±7.56, median 28.14, range 15.36-51.28.

**Figure 1:**
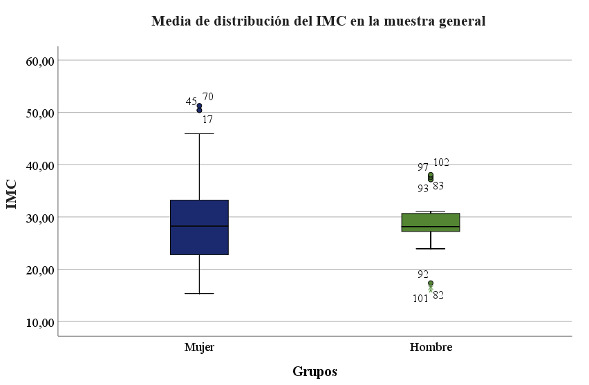
Mean Body Mass Index in Study Groups.

Table 2 and Figure 1 show the distribution of body mass index in the study groups. There were no significant differences observed (p=0.739).

**Table 2:** Body Mass Index in Study Groups.

Group	Mean	SD	Median	Range
Females Group	29.29	8.09	28.24	15.36-51.28
Males Group	28.80	5.96	28.14	15.97-38.09

#### 3.3 Oral Health-Related Quality of Life (OHIP-14Sp).

The mean Oral Health-Related Quality of Life (OHIP-14Sp) score was 13.19, SD ±13.57, median 8.00, range 0-52.

**Figure 2:**
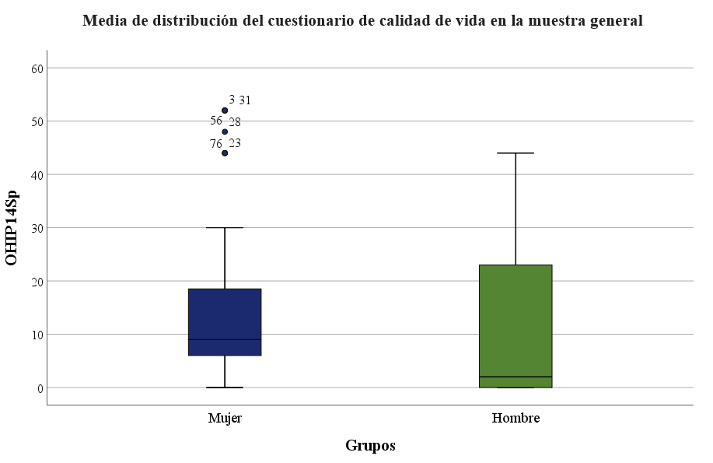
Mean Oral Health-Related Quality of Life (OHIP-14Sp) in the study groups.

Figure 2 and Table 3 display the measurement of Oral Health-Related Quality of Life (OHIP-14Sp) in the study groups, which was higher in the female group with significant differences (p=0.028).

**Table 3:** Oral Health-Related Quality of Life (OHIP-14Sp) in the study groups.

Group	Mean	SD	Median	Range
Female Group	14,15	13,39	9,00	0-52
Male Group	10,55	13,98	2,00	0-44

**Figure 3:**
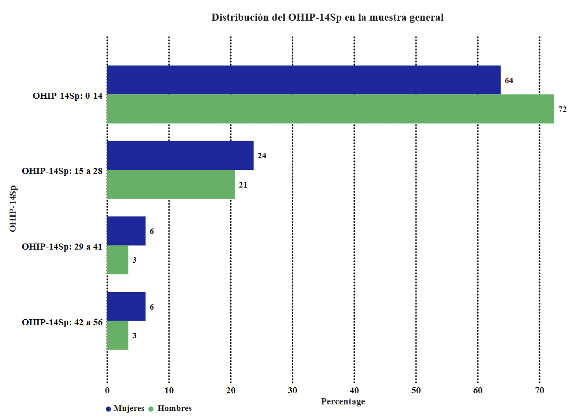
Distribution of Oral Health-Related Quality of Life (OHIP-14Sp) in the study groups.

Table 4 and Figure 3 compare Oral Health-Related Quality of Life (OHIP-14Sp) in the study groups.

**Table 4:** Comparison of Oral Health-Related Quality of Life (OHIP-14Sp) in the study groups.

Groups	Females,		Males,		Total,		
	n= 80		n= 29		n=109		
OHIP-14Sp	N	%	n	%	N	%	p-value
OHIP-14Sp: 0-14	51	63.75	21	72.41	72	66.06	0.4948
OHIP-14Sp: 15 - 28	19	23.75	6	20.69	25	22.94	0.8026
OHIP-14Sp: 29 - 41	5	6.25	1	3.45	6	5.50	1.0000
OHIP-14Sp: 42 - 56	5	6.25	1	3.45	6	5.50	1.0000

#### 3.4. Primary oral issue.

**Figure 4:**
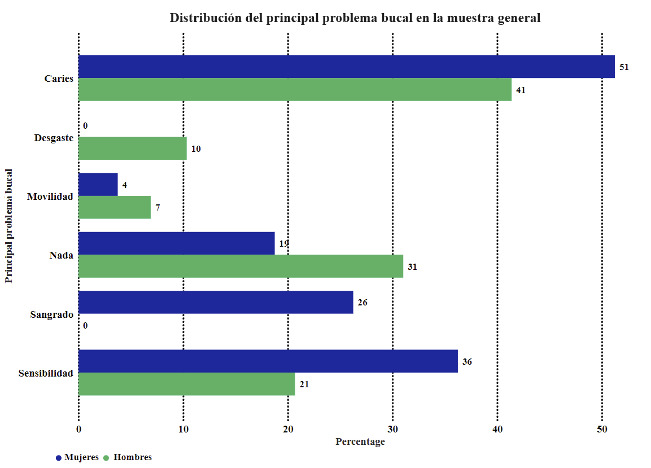
Distribution of the primary oral issue in the study groups.

Table 5 and Figure 4 compare the primary oral issue in the study groups.

**Table 5:** Comparison of the primary oral issue in the study groups.

Groups	Females, n= 80		Males, n= 29		Total, n=109		
Primary oral issue	n	%	n	%	N	%	p-value
Nothing	15	18.75	9	31.03	24	22.02	0.1956
Caries	41	51.25	12	41.38	53	48.62	0.3932
Sensibility	29	36.25	6	20.69	35	32.11	0.1649
Mobility	3	3.75	2	6.90	5	4.59	0.6074
Bleeding	21	2625	0	0.00	21	19.27	0.0008
Wear	0	0.00	3	10.34	3	2.75	0.0174

#### 3.5. Adherence to the Mediterranean diet.

**Figure 5:**
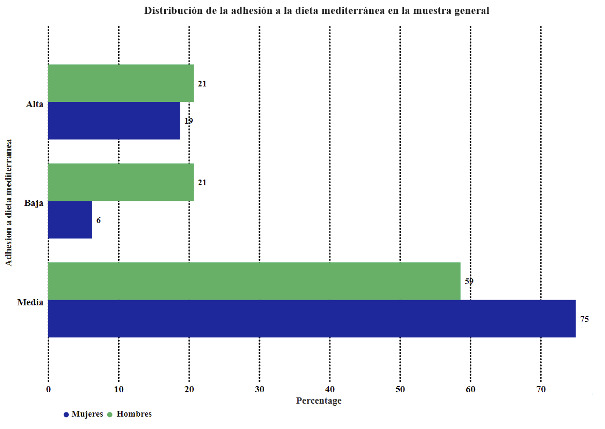
Distribution of adherence to the Mediterranean diet in the study groups.

Table 6 and Figure 5 compare the study groups' adherence to the Mediterranean diet.

**Table 6:** Comparison of adherence to the Mediterranean diet in the study groups.

Groups	Females, n= 80		Males, n= 29		Total, n=109		
Adherence to the Mediterranean diet	n	%	N	%	N	%	p-value
High	15	18.75	6	20.69	21	19.27	0.7899
Medium	60	75.00	17	58.62	77	70.64	0.1520
Low	5	6.25	6	20.69	11	10.09	0.0645

## DISCUSSION

The Oral Health Impact Profile (OHIP) questionnaire comprises 14 questions to assess the limitations and effects of an individual's oral health status, including existing oral pathologies. It aims to evaluate the importance of oral health in a person's quality of life. In the case of obesity type 1, lower scores were obtained compared to obesity types 2 and 3, highlighting how obesity affects an individual’s quality of life and daily performance and influences oral health. For instance, a study by Dibello ^30^ in 2023 observed a correlation between high OHIP scores and eating disorders and obesity in elderly individuals in Italy ^31^ study directly links obesity to poor oral health, with dry mouth being one of the effects. However, research conducted by Tengku in 2021 on 400 obese and normal-weight individuals showed no significant impact of obesity on oral health.

Based on the information above, it is evident that further research is required to fully understand the link between high BMI and poor oral health. However, there is evidence to suggest a definite relationship between the two, and it appears that they impact each other, with an increase in either having adverse effects on an individual ^32^.

This impact is more pronounced as individuals age, with little to no noticeable difference among young people. This could be attributed to the aging process exacerbating the impact of obesity on oral health and the chronic nature of obesity and its duration in relation to oral health. It makes sense as obesity-related inflammation is mild and prolonged, leading to adverse effects over time rather than due to its intensity.

Additionally, the severity of obesity is directly related to its impact on oral health, with higher obesity rates likely linked to more significant discomfort and lower oral health-related quality of life.

The Mediterranean diet is a dietary pattern followed by countries and populations bordering the Mediterranean Sea. This diet is characterized by low consumption of meats and carbohydrates compared to, for example, an American diet and a notable increase in vegetable consumption. The benefits of the Mediterranean diet include better glycemic control, lower triglycerides, and a reduced risk of cardiovascular diseases and other health problems, such as cancer ^33^.

In a recent study, participants completed a validated questionnaire on adherence to the Mediterranean diet with 17 questions with "yes" or "no" responses. Each response scored one point for greater compliance with Mediterranean diet parameters or zero points for lesser compliance, with a maximum score of 17 indicating total adherence to the Mediterranean diet and zero indicating the most remarkable difference from this dietary pattern.

The study results showed that the sample had a mean score of 7.57, a median score of 7, and a few subjects achieving a maximum score of 10. An inverse association was found, with lower adherence to the Mediterranean diet and lower scores linked to a significant increase in BMI and waist circumference. This result supports other studies correlating the Mediterranean diet with life expectancy, quality of life, and obesity. Researchers like the Carbajal group and collaborators have shown how the traditional Mediterranean diet is being adapted to modern times to make it even more efficient regarding current food supply and availability, maintaining and enhancing its health and weight control benefits ^34^. According to research conducted by the Schröder group, adopting the Mediterranean diet can regulate body weight, prevent obesity, and act as a protective factor against both type 1 and type 2 diabetes, with the latter being directly linked to obesity ^35^. A 2019 review also confirms the benefits of the Mediterranean diet in controlling body weight, preventing diseases, and promoting a lifestyle linked to increased life expectancy and quality of life ^36^. The disparity between the lifestyle of the population in the state of Yucatan and the Mediterranean diet standards is significantly associated with the high rates of obesity in the region.

## CONCLUSION

The low adherence of the evaluated population to the Mediterranean diet is associated with a high prevalence of caries and a high body mass index, indicating an opportunity for improvement in quality of life.

It is necessary to conduct studies with a larger population to assess other dietary factors associated with obesity and their impact on oral health status.
